# State of the Art Bowel Management for Pediatric Colorectal Problems: Hirschsprung Disease

**DOI:** 10.3390/children10081418

**Published:** 2023-08-20

**Authors:** Elizaveta Bokova, Ninad Prasade, Sanjana Janumpally, John M. Rosen, Irene Isabel P. Lim, Marc A. Levitt, Rebecca M. Rentea

**Affiliations:** 1 Comprehensive Colorectal Center, Department of Surgery, Children’s Mercy Kansas City, Kansas City, MO 64108, USA; 2 Division of Pediatric Gastroenterology, Hepatology and Nutrition, Children’s Mercy Kansas City, Kansas City, MO 64108, USA; 3 Department of Pediatrics, University of Missouri-Kansas City, Kansas City, MO 64108, USA; 4 Department of Surgery, University of Missouri-Kansas City, Kansas City, MO 64108, USA; 5 Division of Colorectal and Pelvic Reconstruction, Children’s National Medical Center, Washington, DC 20001, USA

**Keywords:** bowel management, Hirschsprung disease, botox, enterocolitis, botulinum toxin, irrigation, obstruction, total colonic aganglionosis, fecal incontinence, enema, laxatives, constipation

## Abstract

After an initial pull-though, patients with Hirschsprung disease (HD) can present with obstructive symptoms, Hirschsprung-associated enterocolitis (HAEC), failure to thrive, or fecal soiling. This current review focuses on algorithms for evaluation and treatment in children with HD as a part of a manuscript series on updates in bowel management. In constipated patients, anatomic causes of obstruction should be excluded. Once anatomy is confirmed to be normal, laxatives, fiber, osmotic laxatives, or mechanical management can be utilized. Botulinum toxin injections are performed in all patients with HD before age five because of the nonrelaxing sphincters that they learn to overcome with increased age. Children with a patulous anus due to iatrogenic damage of the anal sphincters are offered sphincter reconstruction. Hypermotility is managed with antidiarrheals and small-volume enemas. Family education is crucial for the early detection of HAEC and for performing at-home rectal irrigations.

## 1. Introduction

Hirschsprung disease (HD) occurs in 1 in 5000 newborns with a predominance in premature males [1,2]. The main goal of surgery is to treat the functional obstruction inherent to the disease process. After this has been accomplished, postoperative care involves continued good bowel emptying to prevent enterocolitis and to achieve social continence [3].

The outcomes after a pull-through highly depend on the initial pull-through quality [4] and the patient’s anorectal and colonic motility. After an initial pull-through, up to 53% of patients experience obstructive symptoms [5,6,7], up to 37% have Hirschsprung-associated enterocolitis (HAEC) [8,9], 7.5% fail to gain expected weight, and up to 48% have fecal incontinence [5,10,11,12,13], all of which negatively affect the patient’s long-term quality of life (QoL) [5,11,14]. Younger children with HD are more likely to develop depression with an otherwise satisfactory QoL [14], while older patients suffer from emotional distress and limited personal and sexual relationships with a decreased QoL [5,14,15]. This disparity amplifies the importance of long-term management of these patients as the consequences of the disease are not limited to the early postoperative period [15,16]. A patient-centered approach with family inclusion at the time of bowel management can improve QoL [17,18] and lead to better long-term outcomes.

Our goal is to report updates on a bowel management program (BMP) for patients with a pediatric colorectal diagnosis (anorectal malformation, Hirschsprung disease, spinal anomaly, and functional constipation) based on the recent literature [19,20,21]. The current manuscript reviews the anatomic features of patients with HD, management of obstructive episodes, soiling, HAEC, and total colonic HD.

## 2. Methods

A review of the literature published before March 2023 in Medline/PubMed, Google Scholar, Cochrane, and EMBASE databases, including original studies, meta-analyses, randomized controlled trials, and systematic reviews, was performed focusing on manuscripts and books published over the last 5–10 years in English. Search keywords included: “bowel management”, “Hirschsprung disease”, “Botox”, “HAEC”, “enterocolitis”, “botulinum toxin”, “irrigation”, “obstruction”, “total colonic aganglionosis”, “fecal incontinence”, “enema”, “laxatives”, and “constipation”. The reference lists of the retrieved articles were checked for other relevant articles not found during the initial search. Articles providing novel insights or addressing current challenges in the field were prioritized. One hundred-one of the selected articles were included in the current review. The data was reported in a narrative format focusing on the recent updates in the bowel management of patients with HD and used to inform an in-depth, stepwise protocol for bowel management. Due to the large number of abbreviations used in the current manuscript, the list of them is added to facilitate reading.

## 3. Anatomic Considerations

To achieve bowel control, patients with HD require both an intact dentate line and anal sphincters. Ideally, a patient should nutritionally thrive, be continent for stool, and not have any episodes of obstruction or enterocolitis [22]. Unfortunately, not all patients achieve such results. Post-pull-through complications can be divided into two categories: (1) obstruction, which ranges from constipation to more severe manifestations of enterocolitis, and (2) fecal incontinence [22].

## 4. Factors Affecting the Outcomes

Long-term outcomes and the need for bowel management following an HD pull-through are dependent on the timing of the surgery, the length of the aganglionic segment, the technique, and the anatomic success of the pull-through. Down syndrome, present in 5% of cases [23,24], is independently associated with poorer outcomes in HD.

### 4.1. Anatomic Factors

The length of the aganglionic segment impacts the incidence of postoperative enterocolitis and bowel management choices. Some studies report a positive correlation between the length of the segment affected and the incidence of HAEC [12,25,26,27]; however, this statement is controversial [28]. Children with short-segment disease, limited to the rectosigmoid, often struggle with constipation post pull-through. Those with long-segment HD can have loose stools with less colon present to absorb water, and these stools are much harder to control [29]; thus, patients with long-segment HD are more likely to develop fecal incontinence given proper sphincter relaxation. Interestingly, a recent article reported that submucosal nerve thickness of the pulled-through colon does not affect functional outcomes [28].

### 4.2. Age-Related Psychosocial Factors

Psychosocial factors play a significant role in the management of patients with HD. In the neonatal period, fear and uncertainty about their newborn’s future, and lack of social support [17,30,31,32] can overwhelm new parents. Complete and clear communication between providers and new parents should be frequent, and every effort made to provide social and psychological support for caregivers. As the child grows and starts attending school, caregivers often face anxiety due to separation from their children [30,31,32], peer rejection, and behavioral concerns related to toilet training and bowel habits [10,17,33,34]. These can often result in absenteeism and poor performance at school [5,10,17,35,36,37]. It is important to address bullying at school, provide the child and their family with support for toilet training, and enable psychological treatment when needed [17]. Parental engagement and support are crucial for a higher quality of life in patients with HD [18,34,37] to help children overcome the struggles associated with their disease [36].

In adolescence, body dysmorphia, low self-esteem, anxiety, and depression are potential evolving concerns that benefit from psychologist-based evaluation and treatment. This can include bullying prevention, and suicide screening [17]. The social aspects of the disease and the need for medical care at the time of classes can lead to academic difficulties which should be addressed [17]. The patients start to focus on increasing independence and development of disease-related self-efficacy [17], which should be taken into consideration when determining the bowel regimen.

When reaching adult age, the patients start to experience anxiety about personal relationships, sexual function, and reproduction, which are subjects for private discussions with their healthcare provider [15,16,17,38]. The transition of care to adult specialties is covered in a related manuscript [19].

## 5. Management of Obstructive Episodes

**Figure 1:**
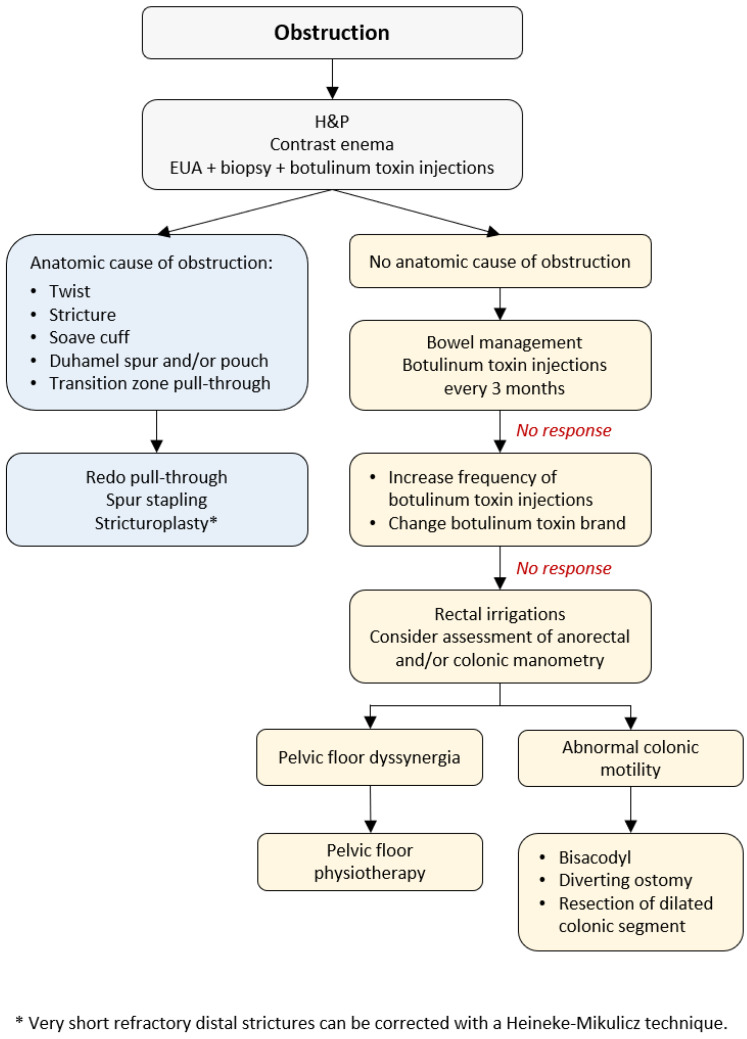
Management of obstructive symptoms following a pull-through. EUA—examination under anesthesia; H&P—history and physical examination.

Abdominal distension, vomiting, failure to thrive, and enterocolitis are the characteristics of obstruction in HD patients reported in 8–53% of cases [6,7]. Some patients can develop obstruction after surgery, while others continue to have issues with obstruction as they did before the pull-through [39]. Severe constipation can result from (1) anatomic cause of obstruction (an anastomotic stricture, obstructing Yancey–Soave cuff, a twist of the pull-through, Duhamel spur or pouch, and transition zone pull-through), or (2) functional causes (internal sphincter achalasia or colonic dysmotility) [40]. The initial evaluation includes obtaining the medical history, physical examination, and a contrast enema to assess the stool burden and rule out a twist of the pull-through (Figure 1). The coloanal anatomy is assessed with a rectal examination under anesthesia (EUA) to rule out an anatomic stricture followed by a full-thickness rectal biopsy to exclude a transition zone pull-through [41], and an empiric botulinum toxin injection to treat non-relaxing sphincters [42]. Care should be taken to identify the presence, or absence, of the dentate line. At our institutions, 100 units of botulinum toxin diluted with 1 mL of normal saline are injected into the internal sphincter circumferentially [43].

### 5.1. Anatomic Causes for Obstruction

**Figure 2:**
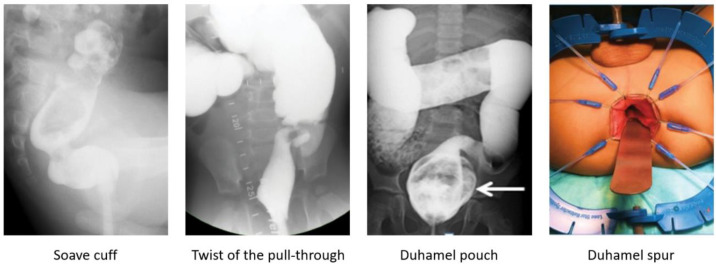
Anatomic causes of obstruction revealed on a contrast enema or examination under anesthesia and requiring surgical correction.

A contrast enema helps to reveal a mechanical obstruction resulting from an anastomotic stricture, obstructing Yancey–Soave cuff, a twist of the pull-through segment, or a Duhamel spur or pouch (Figure 2) [3,39,44]. A rectal biopsy rules out a transition zone pull-through [3,7]. The anatomic causes of obstruction require surgical correction [3,39,44]. An obstructive Soave cuff, a twist of the pull-through, and a mega Duhamel pouch indicate a redo pull-through with the resection of the abnormal colonic segment. A Duhamel spur can be managed with the use of an endovascular stapler to create a common channel between the spur and colon lumen. Should this intervention fail to improve obstructive symptoms, a redo pull-through should be performed [45]. Very short refractory distal strictures can be corrected with a Heineke–Mikulicz technique while a redo pull-through is required for more severe cases.

### 5.2. Nonrelaxing Sphincters

If no anatomic abnormalities are revealed, non-relaxing sphincters are presumed to be the underlying cause of obstruction. Patients with HD lack a rectoanal inhibitory reflex (RAIR) and have no internal anal sphincter relaxation with rectal distension, resulting in obstructive symptoms [46]. As a child grows, they learn to overcome the non-relaxing sphincters [22,39,46] by pushing the stool out with other maneuvers, such as increased intraabdominal pressure and contraction of the abdominal wall musculature [47]. This learning can be facilitated with directed pelvic floor therapy.

As all children with HD have non-relaxing internal anal sphincters, botulinum toxin injections are performed routinely in this patient group. Botulinum toxin injections are effective in 66–72% of patients, usually within 1 month after the injection [42,48], lasting for about 3 months, with positive effects through 6 months [42]. Success is defined as a reduced dosage of laxatives or irrigations required for further bowel management or the absence of any symptoms of obstruction [48]. Wexter et al. reported that 22% of patients did not require further management with laxatives, enemas, or repeat injections after the first botulinum toxin injection [48]. Another study showed more promising results with a 76% improvement rate and 51% of patients achieving good long-term results with laxatives and no further need for rectal enemas [49].

The effect of the botulinum toxin injections is temporary, and a repeat procedure can be required every 3–12 months [42,46,50], with the expectation that the problem will resolve spontaneously with increasing age [39,40,46], commonly by the age of 5 [51], and the injections will become unnecessary [39]. However, Soh et al. emphasized that repeat injections can be associated with a decreased efficacy (44% vs. 77%) [46]. Of note is that in about 9% of patients, injections can lead to temporary, self-limited fecal incontinence [49]. If the botulinum toxin injections do not improve anal sphincter relaxation, increasing the injection frequency or another toxin brand should be considered.

As an alternative to botulinum toxin injections, some authors proposed posterior myectomy to manage nonrelaxing sphincters [52]. However, this procedure is associated with a risk of sphincter damage resulting in permanent fecal incontinence [46,52]. Currently, botulinum toxin injections are the treatment of choice to overcome the sphincter hypertonicity in addition to bowel management with stimulant laxatives, fiber, occasionally stool softeners, or mechanical emptying of the colon with irrigations, rectal enemas, transanal irrigations, or antegrade flushes.

### 5.3. Stepwise Bowel Management

The ability to overcome the non-relaxing sphincters with other defecation maneuvers and the age of the patient determine the optimal bowel regimen. In children with HD, mechanical emptying of the colon (colonic irrigations, rectal enemas, transanal irrigations, and antegrade continence enemas) or medications (laxatives) are utilized. The main differences between the mechanical bowel management options are demonstrated in Table 1.

#### 5.3.1. Rectal Irrigations, Laxatives, and Rectal Enemas

**Figure 3:**
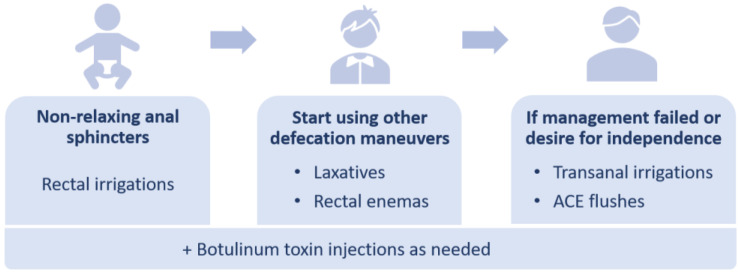
Bowel management based on the patient’s ability to relax the anal sphincters and response to the bowel regimen. ACE—antegrade continence enema.

Before starting a child on a bowel regimen, their age and sphincter relaxation should be considered. Infants and children with persistent hypertonic sphincters retain rectal enema solution in the colon and cannot evacuate the enema due to outlet obstruction, causing nausea and vomiting. For this reason, rectal irrigations are the optimal regimen for this patient group that assists evacuation of the solution (Figure 3). When a child requires minimal sphincter stimulation with the catheter prior to pushing out stool, a switch to rectal enemas may be considered. In children after the age of three, tap water can be used, while in younger patients, only saline is appropriate as tap water can result in hyponatremia and dehydration [53]. Stimulants such as castile soap, glycerin, or bisacodyl can be added to increase colonic contractility. If a child experiences fecal incontinence with stimulant laxatives, an abdominal radiograph assists in determining if the enema is too weak or strong based on the stool burden [53].

As the child grows and learns to expel the stool, with no recurrent episodes of enterocolitis, the regimen can be switched to laxatives or rectal enemas. Initially, a 7-day laxative trial is performed with a daily assessment of the stool burden and clinical symptoms. Stimulant laxatives such as sennosides are utilized to increase colonic contractility and are proven to be effective for the management of constipation in pediatric colorectal patients [54]. Water-soluble fiber can be added as a bulking agent to make the stool bulkier (without constipating), while osmotic laxatives such as lactulose and polyethylene glycol (PEG) 3350 help to make the stool softer if needed [48]. If the laxative trial is ineffective, larger-volume retention enemas are administered to empty the colon mechanically [29,55,56,57]. After successful colon decompression for at least 6–12 months, transition to laxatives can be considered [55].

Importantly, laxatives or enemas should not be given to patients with nonrelaxing sphincters as the stool will accumulate in the distal colon, effectively worsening obstructive symptoms (requiring irrigations to empty and causing nausea and vomiting). In these cases, rectal irrigations are temporarily applied until a botulinum toxin injection is performed to relax the sphincters. Only then can management with laxatives or rectal enemas be continued.

#### 5.3.2. Transanal Irrigations and Antegrade Continence Enemas

If management with medications and/or rectal enemas fail to sufficiently empty the colon, transanal irrigations (TAIs) or antegrade continence enemas (ACEs) are administered (Table 1). TAIs were reported to improve fecal incontinence in patients with HD [58]; however, there is a lack of high-level evidence on the use of TAIs in these children. This regimen is more commonly used in patients with an ARM, functional constipation, or neurogenic bowel.

In patients who fail conservative management or desire to gain more independence, ACE flushes can be administered. Before starting a child on ACEs, it is important to ensure that a rectal enema solution can be adequately expelled. In cases of poor anal sphincter relaxation or poor colonic motility, the ACE solution will be retained, worsening the obstructive symptoms. To improve poor anal sphincter relaxation, botulinum toxin can be injected into the anal sphincter at the time of the ACE procedure to prevent retention of the flush. Postoperatively if a child fails to expel their ACE flush, a catheter is inserted from below to assist in evacuating the solution.

ACEs were reported to successfully treat fecal incontinence in patients with HD [59] resulting in social continence in 54–92% of children [60,61] and a four-time decrease in hospital admissions for disimpactions [60]. Chong et al. reported 50% of patients with HD were successfully weaned off the ACE flushes with 40% of children still using ACE and 1/10 participants having failed ACEs at a median 8.5-year follow-up [62]. Given the misconception of the bowel management strategy in the literature, the generalized rate of success cannot be defined.

#### 5.3.3. Assessment of Anorectal and Colonic Motility

If there are no anatomic causes for obstruction and botulinum toxin injections with bowel management do not improve symptoms, anorectal and/or colonic motility assessment is considered.

Anorectal manometry (AMAN) in patients with HD depicts the defecation dynamics and allows for a diagnosis of pelvic floor dyssynergia which is managed with pelvic floor physiotherapy [20,63]. For further information on the management of this condition, please refer to a related manuscript on functional constipation [64].

Colonic motility is assessed with colonic manometry (CMAN), sitz marker study, or nuclear scintigraphy [65]. In the presence of outlet obstruction, colon motility studies should be interpreted with caution as results may differ when obstruction is relieved. Even though sitz marker study has been proposed as an alternative to CMAN, this method is associated with a higher radiation exposure with numerous clinic visits required for the assessment of the results [66]. Nuclear scintigraphy is available at a limited number of institutions due to its high cost [66]. There are three scenarios of colonic contractility: (1) normal motility, (2) segmental dysmotility, and (3) diffuse dysmotility with no high amplitude propagated contractions (HAPCs) present in the entire colon [65].

Children with normal motility, especially at the toilet-training age, can have persistent constipation and/or soiling due to withholding behavior resulting from excessive pressure on the external sphincter and painful passage of hard stools. This leads to a vicious cycle: the more constipated the child is, the more painful the defecations become [65], and the more likely the child will continue to hold stool. These children require behavioral modifications and stool softeners to ensure an easier and painless passage of stool [67].

Segmental dysmotility can be managed with either a (rare) segmental resection of the dilated segment of the colon [65] or fecal diversion to allow the colon to rest. Patients with no HAPCs throughout the colon have diffuse dysmotility and respond to bisacodyl in 38% of cases [68] or require a diverting ostomy [55].

## 6. Management of Fecal Incontinence

**Figure 4:**
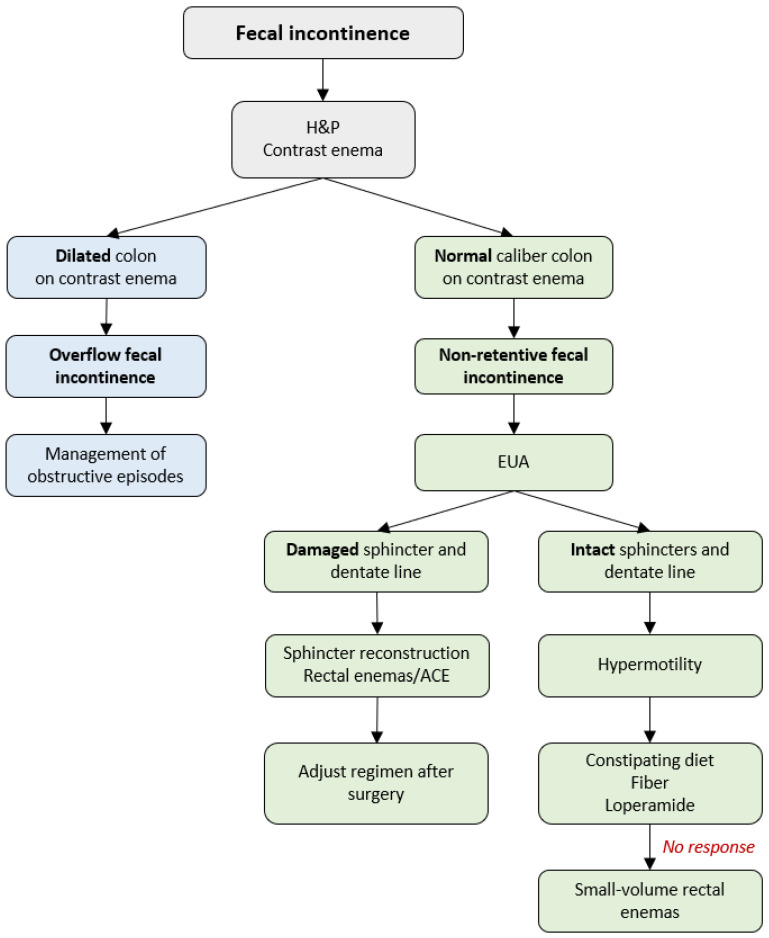
Management of postoperative soiling in patients with Hirschsprung disease. ACE—antegrade continence enema; EUA—examination under anesthesia; H&P—history and physical examination.

Soiling occurs in 3–59% of patients after a pull-through procedure [7,55,69], leading to psychosocial distress [70]. To achieve fecal continence, patients with HD require an intact anal sensation mechanism (dentate line), adequate sphincter tone, and reliable colonic motility. Thus, the underlying causes of postoperative soiling can be (1) overflow fecal incontinence with obstruction being the underlying cause of soiling, or (2) non-retentive fecal incontinence resulting from hypermotility or damage of the anal canal and/or sphincters that occurred at the initial pull-through [40,55]. A stepwise protocol for the diagnosis and treatment of fecal incontinence in patients with HD is demonstrated in Figure 4.

The first step of management is obtaining the medical history, physical examination, and a contrast enema to assess the stool burden (Figure 4). A dilated colon is a mark of overflow fecal incontinence resulting from excessive accumulation of stool in the colon, chronic dilation of the neorectum, and fecal impaction leading to leakage [55]. These patients are managed according to the algorithm for the treatment of obstructive episodes described in the previous section of the current manuscript. A normal caliber colon on the contrast enema defines a non-retentive fecal incontinence with no underlying obstruction and requires a EUA to assess the anatomy and reveal the underlying cause of soiling.

### 6.1. Damaged Anal Sphincters and Dentate Line

In some cases, the dentate line and/or sphincter complex are damaged during the pull-through. These patients’ stooling goals are not met with dietary modification, fiber, and laxatives as they lack the anatomic essentials to achieve voluntary bowel movements. Osmotic laxatives are particularly problematic in these children as a part of the continence mechanism (rectum) is resected as part of the aganglionic colon during the initial pull-through leading to poor detection of stool and fecal incontinence [22,40].

For sphincters that were severely stretched or damaged during the initial pull-through, there was previously no surgical solution other than enemas or ACE. In 2021, a report on sphincter reconstruction for the treatment of fecal incontinence in patients with overstretched sphincters and damaged dentate line during the primary pull-through was proposed. The reconstruction aims to tighten the sphincters around the pull-through to help the patient hold the stool and achieve bowel control [47,71]. At a one-year follow-up in collaboration with gastroenterology motility and colorectal surgery, improvement of the Baylor Continence Scale and bowel control was reported in a small series of patients without neurologic concerns. Half of the patients demonstrated a capacity for voluntary bowel movements, and the others gained independence from their bowel regimen [47]. Additionally, surgical correction of the patulous sphincters and bowel management are required with the goal of maintaining solid stool and consistent emptying of the rectum [47,55].

### 6.2. Intact Anal Canal and Sensation

If the sphincter complex is intact and the sensation is adequate, the patient has a great potential for voluntary bowel movements [40,55] if they are medically managed correctly. In such cases, soiling occurs due to hypermotility characterized by the rapid transit of stool through the colon. The neorectum pulled through at the time of reconstruction for HD is a proximal colonic segment with HAPCs. The continence mechanisms cannot cope with the fast-moving colon connected to the anus resulting in fecal incontinence [68].

**Figure 5:**
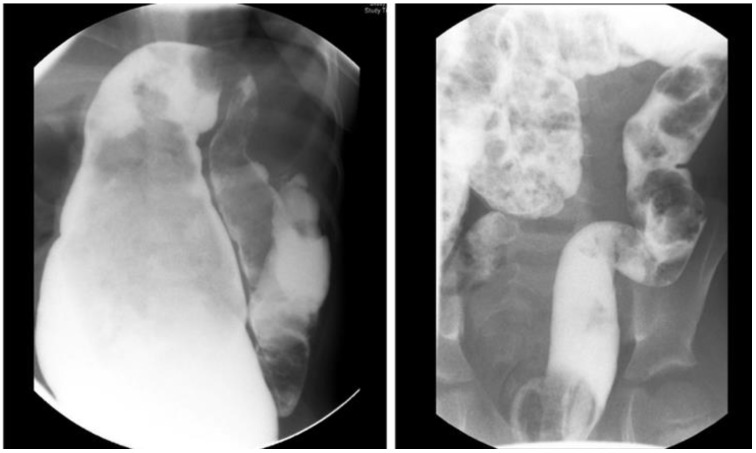
Contrast enema of a hypomotile (**on the left**) and hypermotile (**on the right**) colon. Reprinted from Ref. [55] with permission from Springer Nature.

Hypo- and hypermotility can be differentiated on a contrast enema (Figure 5) [55]. A hypermotile colon typically has normal caliber, while a dilated colon with contrast retention characterizes hypomotility. Colonic manometry can provide additional information on the motility of the bowel [68,72].

Patients with hypermotility can have voluntary bowel movements and are initially managed with constipating diet and fiber [55] to provide bulk stool with antidiarrheals (loperamide, atropine/diphenoxylate) added if needed [40,55]. If soiling persists, small-volume enemas are administered alone or as an addition to the medical management [55,57]. Importantly, laxatives should be avoided in this group of patients as stimulation of the colon or softer stools aggravate fecal incontinence [68].

## 7. Total Colonic Hirschsprung Disease

The optimal timing of a pull-through in patients with total colonic Hirschsprung disease (TCHD) is crucial for functional outcomes. The pull-through is ideally performed between 6–18 months of age if the stool is thick and the patient grows well without requiring formula supplementation or hyperalimentation. Before a pull-through, a trial of stool thickening is performed [73]. Postponing ileostomy closure can lead to stool withholding and proctalgia fugax that, in severe cases, requires ileostomy replacement [74]. The diverted colon segment also remains at risk for developing enterocolitis while awaiting a pull-through.

Postoperatively, patients with TCHD are challenging for pediatric surgeons with hypermotility, skin rash, and failure to thrive being the most common concerns [73]. Diet modifications such as excluding sugar-based and oily food and proton pump inhibitors are used from the early postoperative period with water-soluble fiber and loperamide added as needed [73].

At the time of the 1-month postoperative evaluation, the patient is evaluated for bowel management needs. Those with more than five liquid stools daily are started on water-soluble fiber. If skin excoriation is present despite the use of barrier creams and skin protection, the child is started on loperamide in the dosage of 0.4–0.5 mg/kg [73] to slow the colonic transit and decrease the secretion of gastric acid, biliary, and pancreatic enzymes, and thus reduce the volume of the intestinal lumen [75]. Patients who experience nighttime accidents are prescribed small-volume rectal enemas (100 mL saline) and/or increased doses of loperamide or atropine/diphenoxylate before bedtime. Further follow-up visits or phone calls are scheduled at 3, 6, and 12 months postoperatively [73]. Telemedicine and other organizational aspects of the bowel management program are described in a related article [19].

## 8. Hirschsprung-Associated Enterocolitis

Hirschsprung-associated enterocolitis (HAEC) is a potentially life-threatening complication with a median perioperative prevalence of approximately 18% (range 6–60%) [27]. Recurrent episodes mandate evaluation for possible causes of obstruction [3,39]. The condition is associated with high morbidity and mortality rates of up to 30% [76,77,78,79,80] accounting for most mortality cases associated with HD [80].

HAEC is characterized by abdominal distension, fever, explosive diarrhea, and lethargy (Figure 2) [6,25,81,82], with a more severe presentation in patients with Down syndrome [83,84]. Re-operations for HD are a risk factor for recurrent episodes of HAEC [76]. A history of preoperative enterocolitis can cause a higher rate of HAEC post-pull-through [76,85]; however, this statement is controversial [12]. Another risk factor for postoperative HAEC is a longer aganglionic segment [12,25,26,27].

### 8.1. Prevention and Treatment

The proposed measures for the prevention of postoperative HAEC include routine rectal irrigations and botulinum toxin injections after the pull-through. Irrigations were proven to be an effective preventive measure when initiated 2 weeks post-pull-through for 2–6 months [12,46,86,87]. Botulinum toxin injections are used only for the prevention of HAEC and obstructive symptoms, not its treatment [42]; however, the efficacy and timing of botulinum toxin in relation to HAEC remain controversial. Some authors report the procedure being effective [43,88] with a decreased efficacy if repeat injections are required (44%) [46]. However, other recent studies showed that routine one-month postoperative botulinum toxin injections did not significantly decrease the incidence of HAEC [22,89]. Interestingly, Ahmad et al. reported that patients undergoing postoperative injections developed HAEC sooner when compared with a non-botulinum group (38 vs. 253 days) [22]. However, a recent study with a small subset of patients demonstrated that the incidence of HAEC decreased significantly when botulinum toxin was injected at the time of pull-through [90]. A recent study by Zhang et al. described the placement of a rectal tube immediately after the pull-through and keeping it in the neorectum for 5 days reduced the incidence of HAEC in the early postoperative period and decreased its recurrence in a long-term perspective [91].

**Figure 6:**
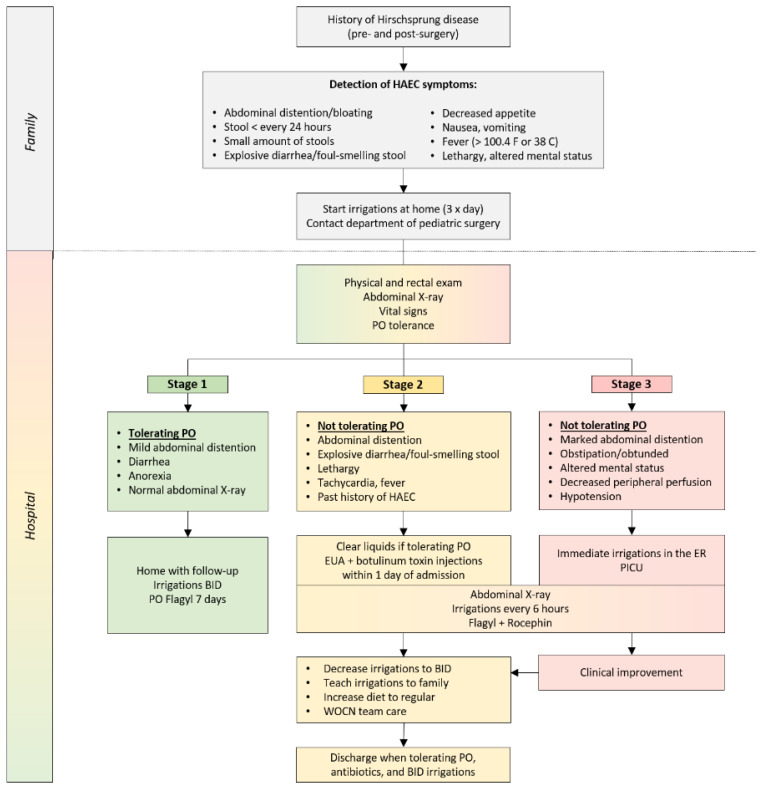
Management of Hirschsprung-associated enterocolitis. BID—twice a day; ER—emergency room; HAEC—Hirschsprung-associated enterocolitis; PICU—pediatric intensive care unit; PO—per oral; WOCN—wound, ostomy, and continence nursing. Modified from Ref. [92] with permission from Springer Nature.

The protocols for HAEC management vary by institution [92]. A sample algorithm for the diagnosis and treatment of enterocolitis is depicted in Figure 6 with family education being a critical component for detecting early symptoms of HAEC and its prevention by performing early irrigations at home [12,93]. Treatment of HAEC includes rectal irrigations, antibiotics administration, and hydration [6,12,83,93]. As the current manuscript is focusing on bowel management of patients with HD, a detailed protocol of HAEC treatment will not be covered in this article and can be reviewed in a recent article by Svetanoff et al. [92].

### 8.2. Education of the Families

As mentioned in the previous section, the education of the caregivers is crucial for the achievement of optimal outcomes and timely detection of complications. While under inpatient care, the parents are taught to irrigate to perform the manipulation at home should any symptoms of enterocolitis occur before the child presents to the emergency room (ER) or surgical clinic. A recent study conducted by Muntean et al. presented medical alert cards that were created for families to facilitate the diagnosis of HAEC [94]. When given out to the families, the cards were shown to increase awareness of HAEC and improve communication between peripheral hospitals and tertiary centers [94].

### 8.3. Rectal (Colonic) Irrigations Technique

**Figure 7:**
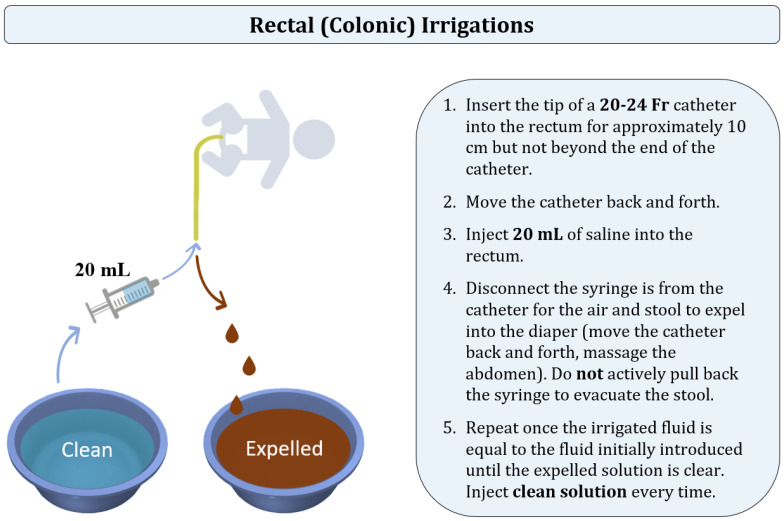
Technique of rectal (colonic) irrigations.

There have been various irrigation techniques described by institutions [95,96,97,98,99,100]. In the current manuscript, we are focusing on the algorithm utilized in the pediatric colorectal centers to which the authors are affiliated. Colonic irrigations, also called rectal irrigations, are performed to facilitate the evacuation of stool and bacteria out of the colon by the introduction of normal saline (Figure 7). The manipulation technique is different from the one in rectal enemas as the goal of irrigations is to actively evacuate the colonic contents by moving the catheter back and forth instead of passively waiting for the patient to expel the solution. In children of three years of age and older, tap water can be used.

Regardless of age, aliquots of 20 mL of saline are used with a 60 mL catheter tip syringe. A 20 to 24 Fr silicone Foley catheter is typically used for irrigations [95]; however, some institutions recommend using a 16 Fr catheter for patients under the age of one year, and 24 Fr in older children [96,99]. A “large bore” silicone catheter can be used instead of a Foley catheter [56]. The tip of the catheter is lubricated and inserted into the rectum for approximately 10 cm but not beyond the end of the catheter. The catheter is gently moved back and forth to facilitate the escape of stool and air. Subsequently, the catheter tip syringe is connected to the end of the catheter, and 20 mL of saline is injected into the rectum through the catheter. Some institutions insert different solution volumes ranging from 10 to 60 mL based on the patient’s age or the institutional protocols [96,98,99]. The syringe is then disconnected from the catheter, allowing the air or stool to expel into the diaper. Catheter insertions are minimized as the catheter gently moves into pockets of air and stool proximally and very distally. The catheter is moved back and forth, and the abdomen is massaged to help with stool evacuation.

Once the irrigated fluid is equal to the fluid initially introduced, the process is repeated with an additional 20 mL of fresh saline at each time until there is an adequate evacuation of stool and the effluent is clear [95,96]. Even though some institutions recommend calculating the quantity of the inserted solution based on the patient’s weight [99], it is crucial to clean the colon and, therefore, make sure that the expelled liquid is clear [96,97,98].

## 9. Outcomes of a Bowel Management Program

In 2020, Kilpatrick et al. published a manuscript on the long-term results of a dedicated BMP in colorectal patients [101]. The study included 41 patients with HD where 86–89% of children were successfully managed with laxatives or rectal enemas at the end of the bowel management week [101]. At a longer follow-up, 45% of patients succeeded at 6 months, and 45% were clean of the bowel regimen at one year [101]. The authors emphasized the importance of regular adjustment of the regimen and consistency with the medications or mechanical emptying of the colon which are the key to the success of a BMP [101].

The outcomes also depend on the institutional approach to bowel management and the care team’s capability to address the child’s individual needs. If necessary, evaluation and management cannot be provided, a referral of the patient to a higher-level center should be considered.

## 10. Conclusions

Patients with HD who have obstructive symptoms, soiling, failure to thrive, or recurrent enterocolitis require a thorough evaluation for underlying anatomic causes including a contrast enema and examination under anesthesia. Children with constipation with no anatomic cause of obstruction and those with overflow fecal incontinence are managed with botulinum toxin injections, laxatives, soluble fiber, osmotic laxatives, or mechanical management. If these treatments fail to improve the symptoms, anorectal or colonic motility assessment helps direct therapy, including pelvic floor physiotherapy, colonic resection, or a diverting ostomy. Patients with fecal incontinence due to damaged sphincters and dentate lines utilize bowel management with enemas and antegrade flushes and may be eligible to consider sphincter reconstruction. Following appropriate workup, children with HD who are found to have a hypermotile colon (approximately 10% of those with rectosigmoid transition zone HD) are treated with dietary modification, water-soluble fiber, antidiarrheals, and small-volume enemas. Family education is critically important for the early detection of enterocolitis and subsequent expedited treatment with at-home irrigations.

## Figures and Tables

**Table 1:** Differences between the mechanical bowel management options: colonic irrigations, rectal enemas, transanal irrigations, and antegrade continence enemas (Malone, Neomalone, cecostomy tube).

	Colonic Irrigation	Rectal Enema	Transanal Irrigation	Antegrade Continence Enema
Catheter inserted via	Rectum	Rectum	Rectum	Surgically created channel ^1^
Direction	Retrograde	Retrograde	Retrograde	Antegrade
Solution injection	Active (manually with a syringe)	Passive ^2^	Active (using a pump system)	Passive ^2^
Solution expulsion	Passive (through the catheter)	By the patient (defecation)	By the patient (defecation)	By the patient (defecation)
Independence	No	No	Yes	Yes

^1^ The channel is created between the abdominal wall and the colon (Malone, Neomalone) or cecum (cecostomy tube). ^2^ “Passive” injection implies using an enema bag and waiting for the solution to gradually enter the colon.

## Data Availability

No new data were created or analyzed in this study. Data sharing is not applicable to this article.

## Abbreviations

ACE	Antegrade continence enema
AMAN	Anorectal manometry
BID	Twice a day
BMP	Bowel management program
CMAN	Colonic manometry
ER	Emergency room
EUA	Examination under anesthesia
H&P	History and physical examination
HAEC	Hirschsprung-associated enterocolitis
HAPC	High amplitude propagated contraction
HD	Hirschsprung disease
HMA	Heineke–Mikulicz anoplasty
PEG	Polyethylene glycol
PICU	Pediatric intensive care unit
PO	Per oral
QoL	Quality of life
RAIR	Rectoanal inhibitory reflex
TAI	Transanal irrigation
TCHD	Total colonic Hirschsprung disease
WOCN	Wound, ostomy, and continence nursing
